# A Quantitative Comparison of Social Interactions of Older Adults Pre-COVID-19 Between the United States and Japan

**DOI:** 10.1093/geroni/igab046.1746

**Published:** 2021-12-17

**Authors:** Mariko Nishikitani

**Affiliations:** Kyushu University Hospital, Medical Information Center, Kyushu University Hospital, Fukuoka, Japan

## Abstract

Using the Study on the Lifestyle and Values of Senior Citizens (The Eighth International Study by the Japan Cabinet Office), the social interactions were assessed in the context of health and life satisfaction of the older adults of the U.S and Japan to confirm the relationship between ICT usage and social interactions. The less social interaction was defined as those who answered that they had no "role in the family," "working," or "social activities such as volunteering." The proportion of less-social interaction people and non-use of ICT increased with age, but the proportions of Japanese were higher than that in Americans. The adjusted odds ratio for non-use of ICT to the risk of isolation of the older adults in Japan was 2.43 (95% CI: 1.59-3.73), but no significant relationship was observed in American older adults. Future research will examine the use of ICT by older adults in each country.

